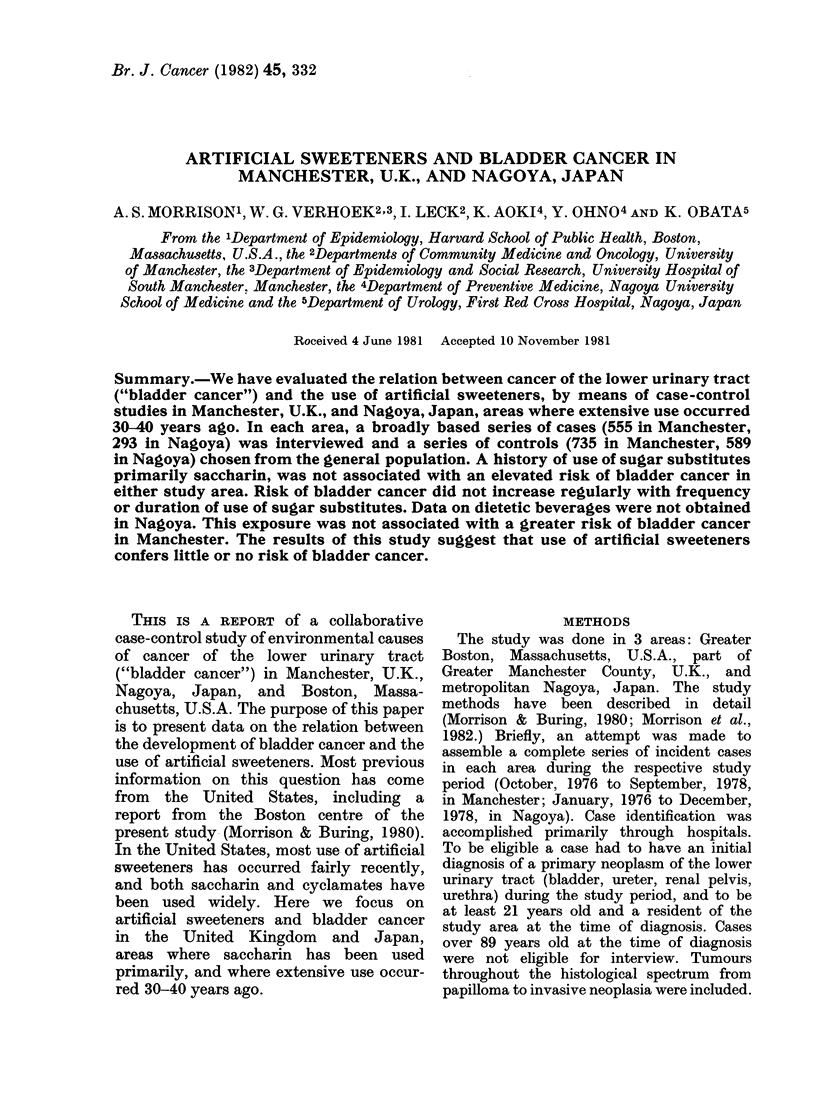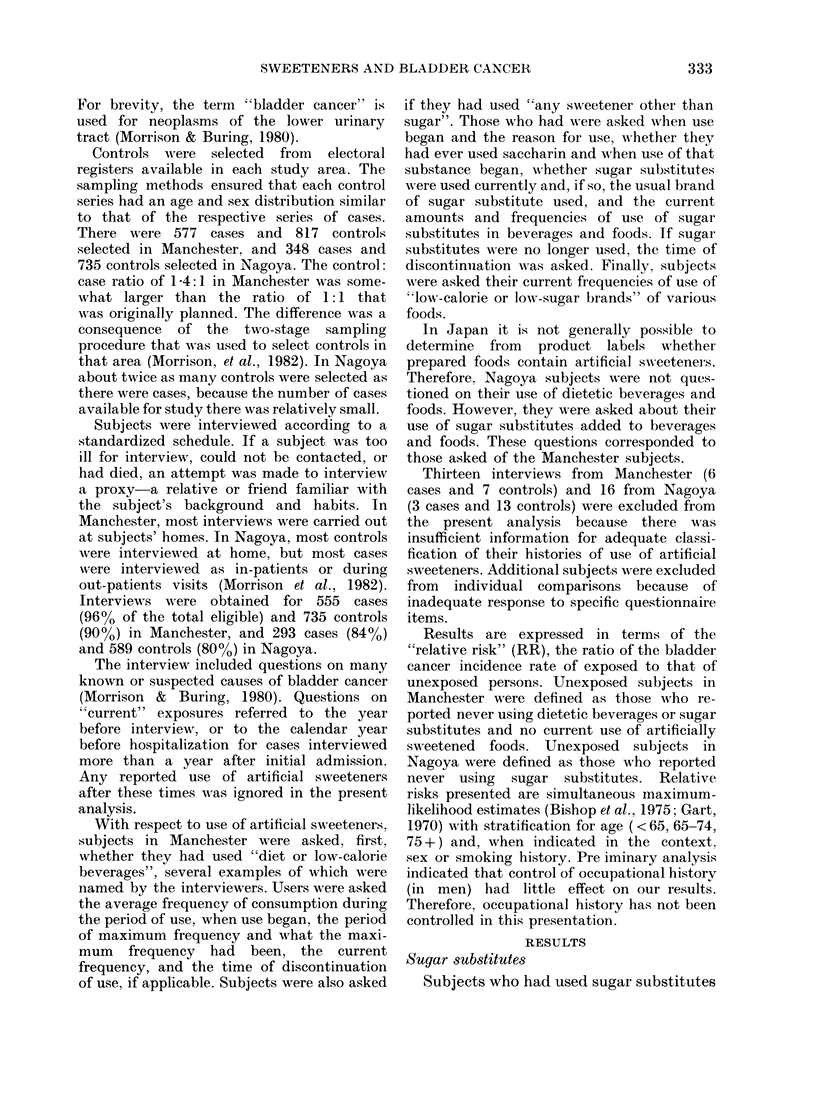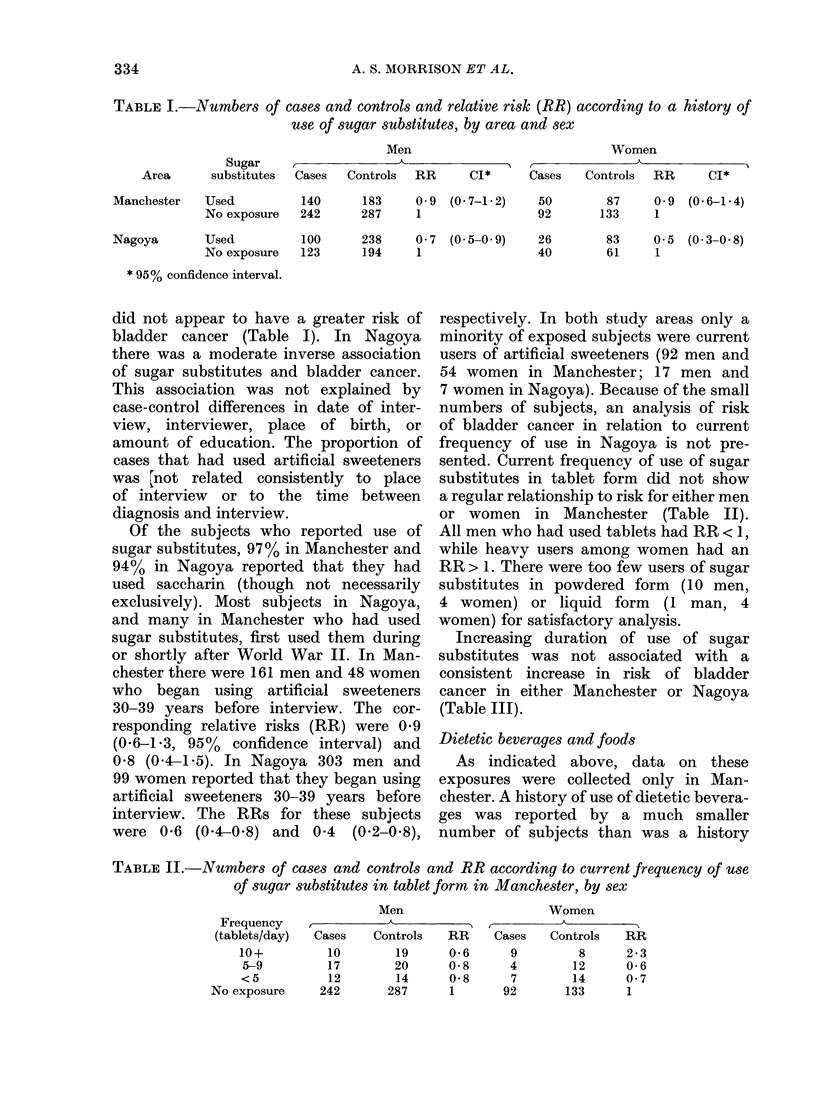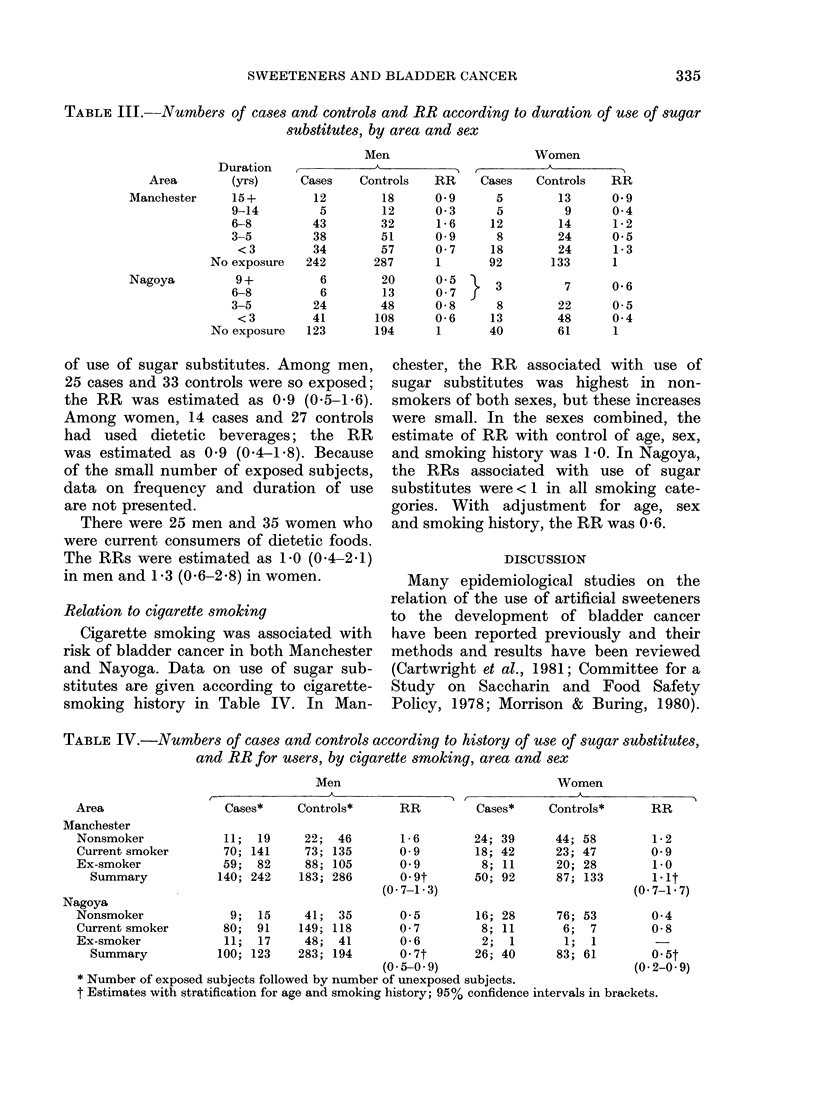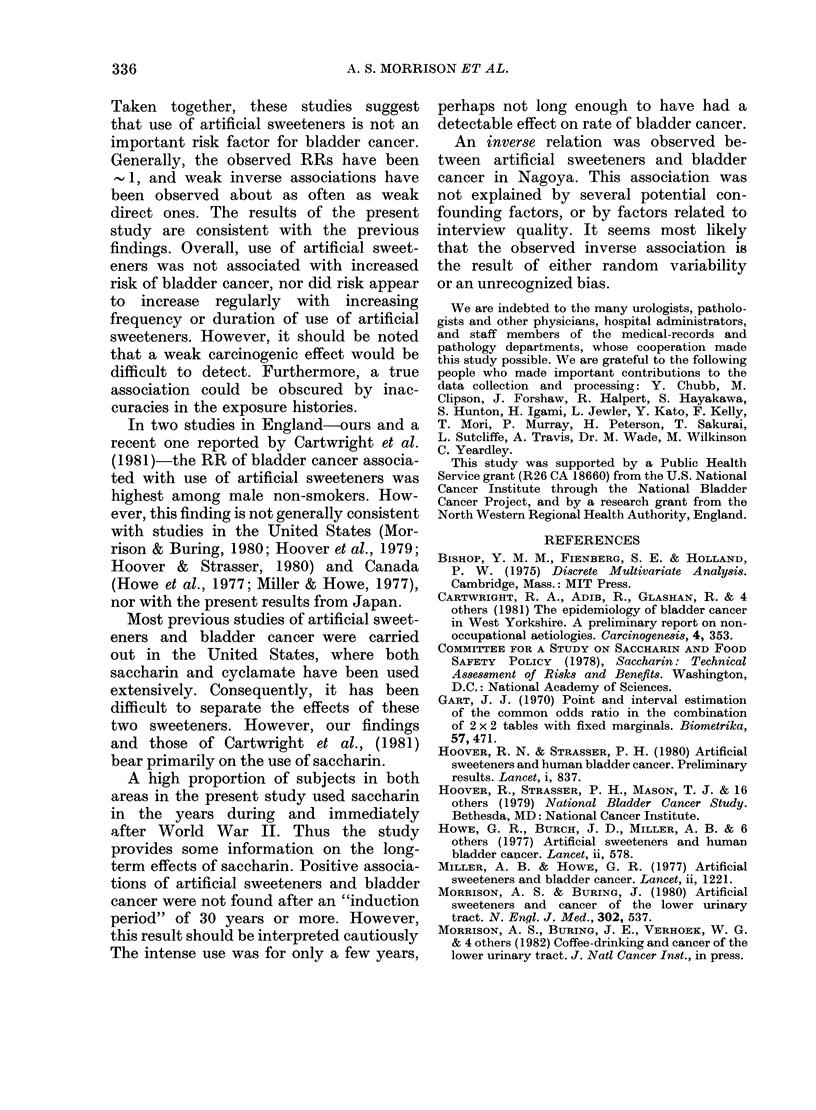# Artificial sweeteners and bladder cancer in Manchester, U.K., and Nagoya, Japan.

**DOI:** 10.1038/bjc.1982.59

**Published:** 1982-03

**Authors:** A. S. Morrison, W. G. Verhoek, I. Leck, K. Aoki, Y. Ohno, K. Obata

## Abstract

We have evaluated the relation between cancer of the lower urinary tract ("bladder cancer") and the use of artificial sweeteners, by means of case-control studies in Manchester, U.K., and Nagoya, Japan, areas where extensive use occurred 30-40 years ago. In each area, a broadly based series of cases (555 in Manchester, 293 in Nagoya) was interviewed and a series of controls (735 in Manchester, 589 in Nagoya) chosen from the general population. A history of use of sugar substitutes primarily saccharin, was not associated with an elevated risk of bladder cancer in either study area. Risk of bladder cancer did not increase regularly with frequency or duration of use of sugar substitutes. Data on dietetic beverages were not obtained in Nagoya. This exposure was not associated with a greater risk of bladder cancer in Manchester. The results of this study suggest that use of artificial sweeteners confers little or no risk of bladder cancer.


Br. J. Cancer (1982) 45, 332

### ARTIFICIAL SWEETENERS AND BLADDER CANCER IN MANCHESTER, U.K., AND NAGOYA, JAPAN

A. S. MORRISON[1], W. G. VERHOEK[2,3], I. LECK[2], K. AOKI[4], Y. OHNO[4] AND K. OBATA[5]

*From the [1]Department of Epidemiology, Harvard School of Public Health, Boston, Massachusetts, U.S.A., the [2]Departments of Community Medicine and Oncology, University of Manchester, the [3]Department of Epidemiology and Social Research, University Hospital of South Manchester, Manchester, the [4]Department of Preventive Medicine, Nagoya University School of Medicine and the [5]Department of Urology, First Red Cross Hospital, Nagoya, Japan*

Roceived 4 June 1981 Accepted 10 November 1981

**Summary.—We have evaluated the relation between cancer of the lower urinary tract ("bladder cancer") and the use of artificial sweeteners, by means of case-control studies in Manchester, U.K., and Nagoya, Japan, areas where extensive use occurred 30–40 years ago. In each area, a broadly based series of cases (555 in Manchester, 293 in Nagoya) was interviewed and a series of controls (735 in Manchester, 589 in Nagoya) chosen from the general population. A history of use of sugar substitutes primarily saccharin, was not associated with an elevated risk of bladder cancer in either study area. Risk of bladder cancer did not increase regularly with frequency or duration of use of sugar substitutes. Data on dietetic beverages were not obtained in Nagoya. This exposure was not associated with a greater risk of bladder cancer in Manchester. The results of this study suggest that use of artificial sweeteners confers little or no risk of bladder cancer.**

THIS IS A REPORT of a collaborative case-control study of environmental causes of cancer of the lower urinary tract ("bladder cancer") in Manchester, U.K., Nagoya, Japan, and Boston, Massachusetts, U.S.A. The purpose of this paper is to present data on the relation between the development of bladder cancer and the use of artificial sweeteners. Most previous information on this question has come from the United States, including a report from the Boston centre of the present study (Morrison & Buring, 1980). In the United States, most use of artificial sweeteners has occurred fairly recently, and both saccharin and cyclamates have been used widely. Here we focus on artificial sweeteners and bladder cancer in the United Kingdom and Japan, areas where saccharin has been used primarily, and where extensive use occurred 30–40 years ago.

### METHODS

The study was done in 3 areas: Greater Boston, Massachusetts, U.S.A., part of Greater Manchester County, U.K., and metropolitan Nagoya, Japan. The study methods have been described in detail (Morrison & Buring, 1980; Morrison *et al.*, 1982.) Briefly, an attempt was made to assemble a complete series of incident cases in each area during the respective study period (October, 1976 to September, 1978, in Manchester; January, 1976 to December, 1978, in Nagoya). Case identification was accomplished primarily through hospitals. To be eligible a case had to have an initial diagnosis of a primary neoplasm of the lower urinary tract (bladder, ureter, renal pelvis, urethra) during the study period, and to be at least 21 years old and a resident of the study area at the time of diagnosis. Cases over 89 years old at the time of diagnosis were not eligible for interview. Tumours throughout the histological spectrum from papilloma to invasive neoplasia were included.

For brevity, the term "bladder cancer" is used for neoplasms of the lower urinary tract (Morrison & Buring, 1980).

Controls were selected from electoral registers available in each study area. The sampling methods ensured that each control series had an age and sex distribution similar to that of the respective series of cases. There were 577 cases and 817 controls selected in Manchester, and 348 cases and 735 controls selected in Nagoya. The control: case ratio of 1·4:1 in Manchester was somewhat larger than the ratio of 1:1 that was originally planned. The difference was a consequence of the two-stage sampling procedure that was used to select controls in that area (Morrison, *et al.*, 1982). In Nagoya about twice as many controls were selected as there were cases, because the number of cases available for study there was relatively small.

Subjects were interviewed according to a standardized schedule. If a subject was too ill for interview, could not be contacted, or had died, an attempt was made to interview a proxy—a relative or friend familiar with the subject's background and habits. In Manchester, most interviews were carried out at subjects' homes. In Nagoya, most controls were interviewed at home, but most cases were interviewed as in-patients or during out-patients visits (Morrison *et al.*, 1982). Interviews were obtained for 555 cases (96% of the total eligible) and 735 controls (90%) in Manchester, and 293 cases (84%) and 589 controls (80%) in Nagoya.

The interview included questions on many known or suspected causes of bladder cancer (Morrison & Buring, 1980). Questions on "current" exposures referred to the year before interview, or to the calendar year before hospitalization for cases interviewed more than a year after initial admission. Any reported use of artificial sweeteners after these times was ignored in the present analysis.

With respect to use of artificial sweeteners, subjects in Manchester were asked, first, whether they had used "diet or low-calorie beverages", several examples of which were named by the interviewers. Users were asked the average frequency of consumption during the period of use, when use began, the period of maximum frequency and what the maximum frequency had been, the current frequency, and the time of discontinuation of use, if applicable. Subjects were also asked if they had used "any sweetener other than sugar". Those who had were asked when use began and the reason for use, whether they had ever used saccharin and when use of that substance began, whether sugar substitutes were used currently and, if so, the usual brand of sugar substitute used, and the current amounts and frequencies of use of sugar substitutes in beverages and foods. If sugar substitutes were no longer used, the time of discontinuation was asked. Finally, subjects were asked their current frequencies of use of "low-calorie or low-sugar brands" of various foods.

In Japan it is not generally possible to determine from product labels whether prepared foods contain artificial sweeteners. Therefore, Nagoya subjects were not questioned on their use of dietetic beverages and foods. However, they were asked about their use of sugar substitutes added to beverages and foods. These questions corresponded to those asked of the Manchester subjects.

Thirteen interviews from Manchester (6 cases and 7 controls) and 16 from Nagoya (3 cases and 13 controls) were excluded from the present analysis because there was insufficient information for adequate classification of their histories of use of artificial sweeteners. Additional subjects were excluded from individual comparisons because of inadequate response to specific questionnaire items.

Results are expressed in terms of the "relative risk" (RR), the ratio of the bladder cancer incidence rate of exposed to that of unexposed persons. Unexposed subjects in Manchester were defined as those who reported never using dietetic beverages or sugar substitutes and no current use of artificially sweetened foods. Unexposed subjects in Nagoya were defined as those who reported never using sugar substitutes. Relative risks presented are simultaneous maximum-likelihood estimates (Bishop *et al.*, 1975; Gart, 1970) with stratification for age (<65, 65–74, 75+) and, when indicated in the context, sex or smoking history. Pre iminary analysis indicated that control of occupational history (in men) had little effect on our results. Therefore, occupational history has not been controlled in this presentation.

### RESULTS

### *Sugar substitutes*

Subjects who had used sugar substitutes

TABLE I.—*Numbers of cases and controls and relative risk (RR) according to a history of use of sugar substitutes, by area and sex*

| Area | Sugar substitutes | Men | | | | Women | | | |
|---|---|---|---|---|---|---|---|---|---|
| | | Cases | Controls | RR | CI* | Cases | Controls | RR | CI* |
| Manchester | Used | 140 | 183 | 0·9 | (0·7–1·2) | 50 | 87 | 0·9 | (0·6–1·4) |
| | No exposure | 242 | 287 | 1 | | 92 | 133 | 1 | |
| Nagoya | Used | 100 | 238 | 0·7 | (0·5–0·9) | 26 | 83 | 0·5 | (0·3–0·8) |
| | No exposure | 123 | 194 | 1 | | 40 | 61 | 1 | |

* 95% confidence interval.

did not appear to have a greater risk of bladder cancer (Table I). In Nagoya there was a moderate inverse association of sugar substitutes and bladder cancer. This association was not explained by case-control differences in date of interview, interviewer, place of birth, or amount of education. The proportion of cases that had used artificial sweeteners was not related consistently to place of interview or to the time between diagnosis and interview.

Of the subjects who reported use of sugar substitutes, 97% in Manchester and 94% in Nagoya reported that they had used saccharin (though not necessarily exclusively). Most subjects in Nagoya, and many in Manchester who had used sugar substitutes, first used them during or shortly after World War II. In Manchester there were 161 men and 48 women who began using artificial sweeteners 30–39 years before interview. The corresponding relative risks (RR) were 0·9 (0·6–1·3, 95% confidence interval) and 0·8 (0·4–1·5). In Nagoya 303 men and 99 women reported that they began using artificial sweeteners 30–39 years before interview. The RRs for these subjects were 0·6 (0·4–0·8) and 0·4 (0·2–0·8), respectively. In both study areas only a minority of exposed subjects were current users of artificial sweeteners (92 men and 54 women in Manchester; 17 men and 7 women in Nagoya). Because of the small numbers of subjects, an analysis of risk of bladder cancer in relation to current frequency of use in Nagoya is not presented. Current frequency of use of sugar substitutes in tablet form did not show a regular relationship to risk for either men or women in Manchester (Table II). All men who had used tablets had RR < 1, while heavy users among women had an RR > 1. There were too few users of sugar substitutes in powdered form (10 men, 4 women) or liquid form (1 man, 4 women) for satisfactory analysis.

Increasing duration of use of sugar substitutes was not associated with a consistent increase in risk of bladder cancer in either Manchester or Nagoya (Table III).

### *Dietetic beverages and foods*

As indicated above, data on these exposures were collected only in Manchester. A history of use of dietetic beverages was reported by a much smaller number of subjects than was a history

TABLE II.—*Numbers of cases and controls and RR according to current frequency of use of sugar substitutes in tablet form in Manchester, by sex*

| Frequency (tablets/day) | Men | | | Women | | |
|---|---|---|---|---|---|---|
| | Cases | Controls | RR | Cases | Controls | RR |
| 10+ | 10 | 19 | 0·6 | 9 | 8 | 2·3 |
| 5–9 | 17 | 20 | 0·8 | 4 | 12 | 0·6 |
| < 5 | 12 | 14 | 0·8 | 7 | 14 | 0·7 |
| No exposure | 242 | 287 | 1 | 92 | 133 | 1 |

TABLE III.—*Numbers of cases and controls and RR according to duration of use of sugar substitutes, by area and sex*

| Area | Duration (yrs) | Men Cases | Men Controls | Men RR | Women Cases | Women Controls | Women RR |
|---|---|---|---|---|---|---|---|
| Manchester | 15+ | 12 | 18 | 0·9 | 5 | 13 | 0·9 |
| | 9–14 | 5 | 12 | 0·3 | 5 | 9 | 0·4 |
| | 6–8 | 43 | 32 | 1·6 | 12 | 14 | 1·2 |
| | 3–5 | 38 | 51 | 0·9 | 8 | 24 | 0·5 |
| | <3 | 34 | 57 | 0·7 | 18 | 24 | 1·3 |
| | No exposure | 242 | 287 | 1 | 92 | 133 | 1 |
| Nagoya | 9+ | 6 | 20 | 0·5 | 3 (9+ and 6–8 combined) | 7 | 0·6 |
| | 6–8 | 6 | 13 | 0·7 | | | |
| | 3–5 | 24 | 48 | 0·8 | 8 | 22 | 0·5 |
| | <3 | 41 | 108 | 0·6 | 13 | 48 | 0·4 |
| | No exposure | 123 | 194 | 1 | 40 | 61 | 1 |

of use of sugar substitutes. Among men, 25 cases and 33 controls were so exposed; the RR was estimated as 0·9 (0·5–1·6). Among women, 14 cases and 27 controls had used dietetic beverages; the RR was estimated as 0·9 (0·4–1·8). Because of the small number of exposed subjects, data on frequency and duration of use are not presented.

There were 25 men and 35 women who were current consumers of dietetic foods. The RRs were estimated as 1·0 (0·4–2·1) in men and 1·3 (0·6–2·8) in women.

*Relation to cigarette smoking*

Cigarette smoking was associated with risk of bladder cancer in both Manchester and Nayoga. Data on use of sugar substitutes are given according to cigarette-smoking history in Table IV. In Manchester, the RR associated with use of sugar substitutes was highest in non-smokers of both sexes, but these increases were small. In the sexes combined, the estimate of RR with control of age, sex, and smoking history was 1·0. In Nagoya, the RRs associated with use of sugar substitutes were < 1 in all smoking categories. With adjustment for age, sex and smoking history, the RR was 0·6.

### DISCUSSION

Many epidemiological studies on the relation of the use of artificial sweeteners to the development of bladder cancer have been reported previously and their methods and results have been reviewed (Cartwright *et al.*, 1981; Committee for a Study on Saccharin and Food Safety Policy, 1978; Morrison & Buring, 1980).

TABLE IV.—*Numbers of cases and controls according to history of use of sugar substitutes, and RR for users, by cigarette smoking, area and sex*

| Area | Men Cases* | Men Controls* | Men RR | Women Cases* | Women Controls* | Women RR |
|---|---|---|---|---|---|---|
| Manchester | | | | | | |
| Nonsmoker | 11; 19 | 22; 46 | 1·6 | 24; 39 | 44; 58 | 1·2 |
| Current smoker | 70; 141 | 73; 135 | 0·9 | 18; 42 | 23; 47 | 0·9 |
| Ex-smoker | 59; 82 | 88; 105 | 0·9 | 8; 11 | 20; 28 | 1·0 |
| Summary | 140; 242 | 183; 286 | 0·9† (0·7–1·3) | 50; 92 | 87; 133 | 1·1† (0·7–1·7) |
| Nagoya | | | | | | |
| Nonsmoker | 9; 15 | 41; 35 | 0·5 | 16; 28 | 76; 53 | 0·4 |
| Current smoker | 80; 91 | 149; 118 | 0·7 | 8; 11 | 6; 7 | 0·8 |
| Ex-smoker | 11; 17 | 48; 41 | 0·6 | 2; 1 | 1; 1 | — |
| Summary | 100; 123 | 283; 194 | 0·7† (0·5–0·9) | 26; 40 | 83; 61 | 0·5† (0·2–0·9) |

* Number of exposed subjects followed by number of unexposed subjects.
† Estimates with stratification for age and smoking history; 95% confidence intervals in brackets.

Taken together, these studies suggest that use of artificial sweeteners is not an important risk factor for bladder cancer. Generally, the observed RRs have been ~1, and weak inverse associations have been observed about as often as weak direct ones. The results of the present study are consistent with the previous findings. Overall, use of artificial sweeteners was not associated with increased risk of bladder cancer, nor did risk appear to increase regularly with increasing frequency or duration of use of artificial sweeteners. However, it should be noted that a weak carcinogenic effect would be difficult to detect. Furthermore, a true association could be obscured by inaccuracies in the exposure histories.

In two studies in England—ours and a recent one reported by Cartwright *et al.* (1981)—the RR of bladder cancer associated with use of artificial sweeteners was highest among male non-smokers. However, this finding is not generally consistent with studies in the United States (Morrison & Buring, 1980; Hoover *et al.*, 1979; Hoover & Strasser, 1980) and Canada (Howe *et al.*, 1977; Miller & Howe, 1977), nor with the present results from Japan.

Most previous studies of artificial sweeteners and bladder cancer were carried out in the United States, where both saccharin and cyclamate have been used extensively. Consequently, it has been difficult to separate the effects of these two sweeteners. However, our findings and those of Cartwright *et al.*, (1981) bear primarily on the use of saccharin.

A high proportion of subjects in both areas in the present study used saccharin in the years during and immediately after World War II. Thus the study provides some information on the long-term effects of saccharin. Positive associations of artificial sweeteners and bladder cancer were not found after an "induction period" of 30 years or more. However, this result should be interpreted cautiously The intense use was for only a few years, perhaps not long enough to have had a detectable effect on rate of bladder cancer.

An *inverse* relation was observed between artificial sweeteners and bladder cancer in Nagoya. This association was not explained by several potential confounding factors, or by factors related to interview quality. It seems most likely that the observed inverse association is the result of either random variability or an unrecognized bias.

We are indebted to the many urologists, pathologists and other physicians, hospital administrators, and staff members of the medical-records and pathology departments, whose cooperation made this study possible. We are grateful to the following people who made important contributions to the data collection and processing: Y. Chubb, M. Clipson, J. Forshaw, R. Halpert, S. Hayakawa, S. Hunton, H. Igami, L. Jewler, Y. Kato, F. Kelly, T. Mori, P. Murray, H. Peterson, T. Sakurai, L. Sutcliffe, A. Travis, Dr. M. Wade, M. Wilkinson C. Yeardley.

This study was supported by a Public Health Service grant (R26 CA 18660) from the U.S. National Cancer Institute through the National Bladder Cancer Project, and by a research grant from the North Western Regional Health Authority, England.

### REFERENCES

Bishop, Y. M. M., Fienberg, S. E. & Holland, P. W. (1975) *Discrete Multivariate Analysis.* Cambridge, Mass.: MIT Press.

Cartwright, R. A., Adib, R., Glashan, R. & 4 others (1981) The epidemiology of bladder cancer in West Yorkshire. A preliminary report on non-occupational aetiologies. *Carcinogenesis*, **4**, 353.

Committee for a Study on Saccharin and Food Safety Policy (1978), *Saccharin: Technical Assessment of Risks and Benefits.* Washington, D.C.: National Academy of Sciences.

Gart, J. J. (1970) Point and interval estimation of the common odds ratio in the combination of 2 x 2 tables with fixed marginals. *Biometrika*, **57**, 471.

Hoover, R. N. & Strasser, P. H. (1980) Artificial sweeteners and human bladder cancer. Preliminary results. *Lancet*, i, 837.

Hoover, R., Strasser, P. H., Mason, T. J. & 16 others (1979) *National Bladder Cancer Study.* Bethesda, MD: National Cancer Institute.

Howe, G. R., Burch, J. D., Miller, A. B. & 6 others (1977) Artificial sweeteners and human bladder cancer. *Lancet*, ii, 578.

Miller, A. B. & Howe, G. R. (1977) Artificial sweeteners and bladder cancer. *Lancet*, ii, 1221.

Morrison, A. S. & Buring, J. (1980) Artificial sweeteners and cancer of the lower urinary tract. *N. Engl. J. Med.*, **302**, 537.

Morrison, A. S., Buring, J. E., Verhoek, W. G. & 4 others (1982) Coffee-drinking and cancer of the lower urinary tract. *J. Natl Cancer Inst.*, in press.